# A simple method for estimating genetic diversity in large populations from finite sample sizes

**DOI:** 10.1186/1471-2156-10-84

**Published:** 2009-12-16

**Authors:** Stanislav Bashalkhanov, Madhav Pandey, Om P Rajora

**Affiliations:** 1 Canada Research Chair in Forest and Conservation Genomics and Biotechnology, Canadian Genomics and Conservation Genetics Institute, University of New Brunswick, Faculty of Forestry and Environmental Management, 28 Dineen Drive, Fredericton, NB, E3B 6C2, Canada

## Abstract

**Background:**

Sample size is one of the critical factors affecting the accuracy of the estimation of population genetic diversity parameters. Small sample sizes often lead to significant errors in determining the allelic richness, which is one of the most important and commonly used estimators of genetic diversity in populations. Correct estimation of allelic richness in natural populations is challenging since they often do not conform to model assumptions. Here, we introduce a simple and robust approach to estimate the genetic diversity in large natural populations based on the empirical data for finite sample sizes.

**Results:**

We developed a non-linear regression model to infer genetic diversity estimates in large natural populations from finite sample sizes. The allelic richness values predicted by our model were in good agreement with those observed in the simulated data sets and the true allelic richness observed in the source populations. The model has been validated using simulated population genetic data sets with different evolutionary scenarios implied in the simulated populations, as well as large microsatellite and allozyme experimental data sets for four conifer species with contrasting patterns of inherent genetic diversity and mating systems. Our model was a better predictor for allelic richness in natural populations than the widely-used Ewens sampling formula, coalescent approach, and rarefaction algorithm.

**Conclusions:**

Our regression model was capable of accurately estimating allelic richness in natural populations regardless of the species and marker system. This regression modeling approach is free from assumptions and can be widely used for population genetic and conservation applications.

## Background

Accurate estimation of genetic diversity parameters in large natural populations using finite sample sizes is one of the central issues in population and conservation genetic studies and applications. Small sample sizes can lead to significant errors in estimating the genetic diversity of the species in question. For effective genetic resource conservation, sufficient allelic richness and a minimum number of carriers for each allele must be present in the conservation population to ensure its self-sufficiency over generations, otherwise its entire purpose may be compromised if the sampling criteria are not met [1]. This aspect is often overlooked when conservation programs are developed and minimum viable population sizes are determined [2].

Allelic diversity (richness) is one of the most important and commonly used estimators of genetic diversity in populations. It strongly depends on the effective population size and past evolutionary history [3]. However, the number of observed alleles and their frequency distribution also depend on the sample size and the genetic marker system used. Thus, a practical method for reliable estimation of genetic diversity parameters in large populations is needed for population genetic studies and to develop scientifically sound strategies for genetic resource conservation.

Based on the probability theory alone, one can calculate the sample size required to detect alleles with a certain threshold frequency [4,5]. Rarefaction [3] and repeated random subsampling [6] are increasingly popular methods for standardizing the allelic richness for unequal sample sizes. However, there are several possible limitations in using these approaches. First, many estimates are based on the ideal population model. Most temperate and boreal species have experienced tremendous migrations and disturbances since the last glacial maximum. The northernmost populations are evolutionary young and remain dynamic, showing significant deviations from the equilibrium state. Second, distribution of allele frequencies strongly varies among species and marker types. Third, due to the non-linear relationship between sample size and observed allelic richness, simple extrapolation beyond the maximum sample size may not be feasible [6]. Bayesian approaches have also been introduced, but they still cannot predict the allelic richness in large populations when the sample sizes are limited [7].

It is rarely possible to know the true number of alleles in a population unless the entire population can be analyzed, in which case the concept of "sample" is not applicable anymore [8,9]. Theoretically, the effective number of alleles (*m*_*e*_) found in an ideal population can be approximately described as(1)

where *N*_*e *_is the effective population size and *μ *is the mutation rate [10]. When *N*_*e *_→ ∞, the error in *m*_*e *_approaches zero and the parameter *θ *= 4*N*_*e*_*μ *is constant. Ewens in his fundamental work [10] indicated that the distribution of the allele frequencies in a population strongly depends on *θ*. Furthermore, he pointed out that the expected number of alleles *E(k) *for a given sample size *n *can be expressed as(2)

or, for large *n *it can be further simplified [11] to(3)

Estimating the parameter *θ *in a natural population is still complicated: i) in the expression *θ *= 4*N*_*e*_*μ*, there is no or very little information on the mutation rates in plant populations, plus the observed mutation rates are locus-specific, and can be confounded by selection and migration; ii) the effective population size (*N*_*e*_) can be estimated by the coalescent approach [12,13], but the inferences depend on the underlying population genetic model, and the related assumptions may not hold true for the real natural population in question. For nucleotide sequences, Nei introduced nucleotide diversity *ϕ *as another estimator of 4*N*_*e*_*μ *[14], but it is locus-specific and sensitive to the sample size.

Although the Ewens sampling formula and coalescent approach provide theoretical expectations for the allelic richness in a given sample, they normally assume an ideal random mating population of constant size, and without migration and selection. However, natural populations rarely conform to these and other ideal population assumptions. Selection effects may be heterogeneous in time and space, and are extremely difficult to realistically model. Random mating may be hampered by spatial genetic structure and selfing [15,16]. A simple, assumption-free and robust method is needed for estimating allelic diversity in large natural populations. Here, we introduce a simple and robust approach to estimate the genetic diversity in large natural populations based on the empirical finite sample data.

## Methods

### Model development

We investigated several empirical data sets published for a wide variety of plant and animal species to understand the relationship between allelic richness and sample sizes. From the data published for a wide variety of organisms, our own experimental results and computer simulations, we found that the number of alleles observed in a given sample is approximately proportional to the logarithm of the sample size, and the logarithm base depends on the species and the marker system used. Based on these observations, we developed a non-linear regression model to predict the observed allelic richness in a given sample. The model could be defined as:(4)

where *A *is the observed mean number of alleles per locus (allelic richness). The logarithm base *β*_*S *_depends on the species and the marker set used, and *β*_*n *_and *β*_*A *_are the regression coefficients for the sample size and allelic richness, respectively, which depend on the species and molecular markers used. As natural logarithms (ln) are commonly used, we replace the logarithm base to *e*. Thus, equation (4) can be written as follows:(4a)

To further simplify (4a), we introduce the variable *ρ *= 1/ln *β*_*S*_, so the equation (4a) can be written as(5)

The coefficients in the regression model (5) can be empirically determined using the modified random resampling procedure and non-linear regression analysis as described below. At large sample sizes, the coefficient *β*_*n *_becomes negligible, and the equation (5) can be further simplified as(5a)

The empirically derived equation (5a) is similar to the modified Ewens sampling formula in equation (3).

### Model validation and comparison with other methods

We tested the regression model (5) using (i) large empirical data sets for four conifer tree species with contrasting population genetic characteristics, and (ii) simulated population genetic data sets created using Markov-chain-based algorithm with different inherent migration and selfing rates.

Empirical data comprised multilocus genotype data sets for four conifer tree species with contrasting mating systems and inherent genetic diversity levels: microsatellite genotype data for eastern white pine - *Pinus strobus *[8], white spruce - *Picea glauca *[17], red spruce - *Picea rubens *(Bashalkhanov and Rajora, in preparation), and eastern white cedar - *Thuja occidentalis *(Pandey and Rajora, in preparation) were used (Table 1). Microsatellite markers are currently the most popular genetic markers for population and conservation genetics studies. The number of alleles for microsatellites varies greatly among loci and species. This variation provides an ideal but challenging case to develop an appropriate model to determine adequate sample sizes to minimize the effect of sampling error.

**Table 1:** Allelic richness estimated by regression, coalescent and rarefaction

Species	ID	Source data set			Estimated allelic richness				
		
		No. of loci	*N*	A	Subsampling (*n *= 120)	*ρ* (*n *= 120)	*θ*_Ewens_ (*n *= 120)	*θ*_coalescent_ (*n *= 120)	Rarefaction (*n *= 120)
Microsatellites									

*Picea rubens*	PR1	6	180	13.00	11.06	11.04	11.98	9.23	10.68
	PR2	6	180	13.33	11.18	11.17	12.29	8.94	10.71
	PR3	6	180	15.33	12.48	12.44	14.13	11.92	12.19
	PR4	6	180	14.83	12.48	12.44	13.67	12.13	11.92

*Picea glauca*	PG1	6	105	22.83	21.13	21.30	23.49	35.74	20.96
	PG2	6	105	22.83	20.55	20.62	23.49	51.84	20.44

*Pinus strobus*	PS1	13	102	9.77	9.03	9.13	10.11	17.57	9.03
	PS2	13	102	9.23	8.67	8.73	9.55	15.91	8.68

*Thuja occidentalis*	TO1	6	100	7.83	7.18	7.17	8.14	12.26	7.17
	TO2	6	100	9.67	8.95	9.00	10.05	16.28	9.09
	TO3	6	100	8.83	7.86	7.95	9.18	14.06	7.95

Allozymes									

*Pinus strobus*	PS1	15	95	3.20	2.97	2.98	3.38	3.34	2.93
	PS2	15	95	3.27	3.09	3.10	3.59	4.15	3.04

Subsampling - allelic richness estimated by repeated random subsampling in pseudosimulated population data sets based on the empirical data. *ρ *- allelic richness predicted by the regression model (5). *θ*_Ewens _- Allelic richness predicted by the Ewens sampling formula (3), where *θ *was directly calculated from the empirical data set. *θ*_coalescent _- Allelic richness predicted by the Ewens sampling formula (3), where *θ *was estimated by coalescent approach from the empirical data set. Rarefaction - Allelic richness predicted by rarefaction of the source empirical data set to the sample size of *n *= 120.

An additional data set for allozyme markers for eastern white pine was also analyzed. Allozymes have been extensively used in population and conservation genetic studies before the advent of microsatellite markers. Although other markers, such as RAPD (random amplified polymorphic DNA), and AFLP (amplified fragment length polymorphism) have been used in population genetic studies, these markers are not well suited for such studies and have fallen out of favour, primarily due to their diallelic and dominant nature. Codominant SNP (single nucleotide polymorphism) markers are being used in population genetic studies. However, most of them also suffer from the limitation of being diallelic. Since the objective of the present study was to predict the allelic richness in large populations, we used microsatellite and allozyme markers for validating our model, since these markers are codominant and multiallelic.

*Pinus strobus *and *Picea glauca *are predominantly outcrossing species - average multilocus outcrossing rates (*t*_m_) are 0.924, and 0.940, respectively [18,19], and *Picea rubens *and *Thuja occidentalis *are mixed-mating selfing-tolerant species - *t*_m _= 0.595, and 0.635, respectively [20,21]. Samples were collected in natural populations. In *Picea rubens *and *Picea glauca *stands, trees were randomly selected with minimum spacing of 30-50 m between the trees to avoid the possible family structure effects. In the *Pinus strobus *and *Thuja occidentalis *stands, all mature trees within the population were sampled. The number of individuals sampled per population varied from 95 to 180 (Table 1). The number of microsatellite loci used ranged from 6 to 13 (typically employed for population genetic studies). Although the *Pinus strobus *populations were genotyped for 54 allozyme loci [9], we used data for 15 most polymorphic loci to validate our model.

The allelic richness estimates predicted by our regression model were compared with the Ewens sampling formula, coalescent approach, and rarefaction algorithm predictions. Since the experimental data sets had only up to 180 individuals per population, pseudo-simulation data sets of ~10,000 individuals per population were created for each of the four conifer species from their empirical genotype data (Table 1) to address the collection of finite samples from a large natural population. This was done by randomly replicating each genotype within population equal number of times until a population size of ~10,000 was reached, so the resulting data sets had the same distribution of allele frequencies as the original populations. Then random sampling was applied to create test subsamples of 15, 25, 35, 45, 60, 90, and 120 individuals in 50 replicates, and the mean number of alleles per locus was calculated for each sample size. Computations were performed using a Visual Basic program for Microsoft Excel.

The resulting allelic richness values were used to derive the estimates of *ρ *and the *β *coefficients in equation (5) using the Gauss-Newton method implemented in the NLIN procedure in the SAS 9.1.3 statistical package (SAS Institute, Cary, NC). An example of the input data and SAS NLIN output, showing derivation of the regression coefficients (*ρ*, *β*_*n*_, and *β*_*A*_) in (5), is provided in the Additional file 1.

We also tested the simplified Ewens formula (3) as a predictor for allelic richness. First, *θ *was calculated from the allelic richness values observed in the source data sets for natural populations (Table 1) using the modified Ewens formula (3). Then the resulting *θ *was used in equation (3) to estimate the predicted allelic richness at various sample sizes. We also estimated the *θ *values for the source data sets obtained from natural populations using the popular maximum likelihood coalescent approach implemented in the MIGRATE 3.0 program [22,23]. The resulting *θ *estimates were used in the equation (3) to calculate the allelic richness for various sample sizes as indicated above. Also, we estimated the predicted allelic richness values (at *n *= 120) in our experimental samples using the rarefaction procedure implemented in the HP-RARE 1.0 program [24].

Additionally, to estimate the effects of sample size on the observed genetic diversity and genetic subdivision parameters, we calculated the observed and expected heterozygosity, Shannon information index, and F_*ST *_for *Picea rubens *and *Pinus strobus *data sets using the GENALEX 6.1. program [25].

To validate our model, we created 10 artificial data sets each containing 2 populations of 10,000 individuals, with selected combinations of inherent migration and selfing rates, using the Markov chain-based simulation algorithm implemented in the EASYPOP 2.1 program [26]. Migration rates (Nm) were set at 0, 1, 10, 50 and 100 migrants per generation, and selfing (s) was set at 0, 0.2, 0.6, and 0.99 to cover a wide range of mating system and gene flow scenarios. Different combinations of migration and selfing rates would approximate possible deviations from the ideal population model for a wide variety of organisms. High degrees of selfing are not unusual in many mixed-mating selfing-tolerant conifer species, such as *Thuja occidentalis *[20], and extensive gene flow is generally observed in natural plant populations [27]. Mutation rates were set to 0.0002, with the K-allele mutation model implied, and all loci had 20 possible allelic states - these parameters are typical for microsatellite markers [28]. The population size was set constant at *n *= 10,000 to represent a typical large natural plant population. The initial allele states were assigned randomly, and then populations were allowed to evolve under the above-mentioned evolutionary scenarios for 20,000 generations to yield the data set A. Then a sample of *n *= 200 individuals (close to *n *= 180 in experimental populations of *P. rubens*) was taken from the resulting population, and randomly replicated 50 times as described above to create the data set B of *n *= 10,000 individuals. Then repeated random subsampling was performed on both data sets A and B (for *n *= 15, 25, 35, 45, 60, 90, 120, 500, 2,000, 5,000), and the allelic richness was calculated in 50 replicates as described above. Various combinations of Nm and selfing parameters used are provided in the Additional File 2.

## Results and discussion

**Figure 1:**
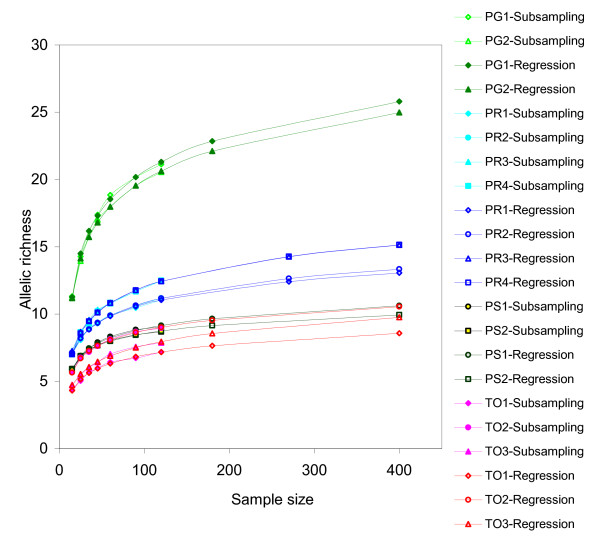
**Allelic richness predicted by subsampling and regression modeling for microsatellite data**. PR: *Picea rubens*, TO: *Thuja occidentalis*, PG: *Picea glauca*, PS: *Pinus strobus*. The population names are provided in Table 1. PG1-Subsampling - TO3-Subsampling: allelic richness estimated by repeated random subsampling from the amplified empirical data. PG1-Regression - TO3-Regression: regression curves for allelic richness predicted by equation (5). Regression curves correspond well with the allelic richness estimates obtained from subsampling of the actual data. Subsampling was performed by replicating the empirical data set up to *n *= 10,000, and randomly drawing samples of a given *n *from the amplified population, in 50 replicates.

**Figure 2:**
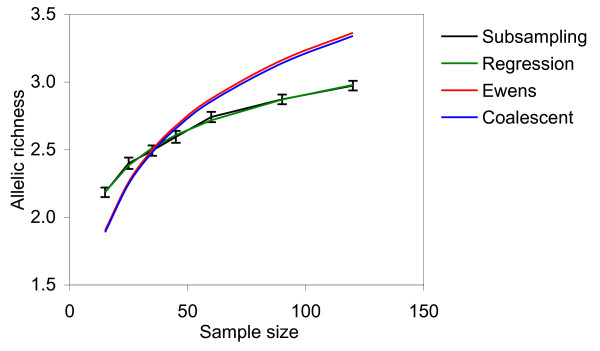
**Allelic richness predicted for one *Pinus strobus *population (PS1) from allozyme data**. Subsampling - allelic richness estimated by repeated random subsampling of the amplified empirical data set in 50 replicates (95% confidence intervals are provided). Regression - allelic richness predicted by equation (5). Ewens - allelic richness predicted by equation (3), *θ *calculated from the source data set. Coalescent - allelic richness predicted by equation (3), *θ *estimated by coalescent from the empirical source data.

**Figure 3:**
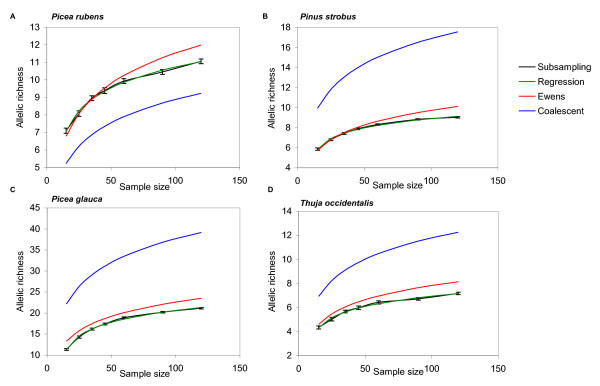
**Allelic richness predicted for selected populations of four species from microsatellite data**. Subsampling - allelic richness estimated by repeated random subsampling (95% confidence intervals are provided). Regression - allelic richness predicted by equation (5). Ewens - allelic richness predicted by equation (3), *θ *calculated from the source data set. Coalescent - allelic richness predicted by equation (3), *θ *estimated by coalescent from the experimental data. **A**: *Picea rubens *population PR1; **B**: *Pinus strobus *population PS1; **C**: *Picea glauca *population PG1; **D**: *Thuja occidentalis *population TO1.

The allelic richess values estimated by the regression model (5), subsampling of the pseudosimulated data sets, and other methods for four conifer species are provided in Table 1, Figure 1, Figure 2, Figure 3, and the Additional File 3. Observed allelic richness gradually increased with the sample size, as expected (Figure 1). The allelic richness values predicted from the equation (5) were in good agreement with those observed in the subsamples of various sizes, and the overall allelic richness in the source populations (Figure 1; Table 1). Ewens and rarefaction allelic richness estimates at *n *= 120 were close to the real observed values, but these methods did not provide consistent results at larger sample sizes (see below). Coalescent approach did not provide reliable estimates for allelic richness. Although the absolute allelic richness values varied among the species and marker systems used, the model developed in this study worked equally well for both predominantly outcrossing and mixed-mating selfing-tolerant species and both types of markers. Allelic richness estimates based on *θ *calculated using the simplified Ewens formula (3) did not provide a good fit with the experimental data - the predicted values were outside of the 95% confidence interval (Figure 2, Figure 3). The coalescent-based *θ *used in the Ewens formula (3) consistently overestimated or underestimated the allelic richness, depending on the species and the marker type (Figure 2; Figure 3; Additional File 3). For example, allelic richness for microsatellite markers was significantly underestimated by the coalescent approach in *Picea rubens *(Figure 3A), and overestimated in *Pinus strobus*, *Picea glauca*, and *Thuja occidentalis *(Figure 3B, Figure 3C, and Figure 3D, respectively). The allozyme allelic richness in *Pinus strobus *was overestimated by this method at larger sample sizes (*n *> 60). At the same time, the allelic richness estimates calculated by our regression model (5) were in good agreement with the experimental data sets for all four species and both marker systems used. Thus, our model was a better predictor for the allelic richness in natural populations as compared to the Ewens formula (Figure 2; Figure 3; Additional File 3).

As mentioned above, the empirically derived equation (5a) is similar to the modified Ewens sampling formula (3). Allelic richness estimates predicted by the Ewens formula (3) significantly deviated from the empirical estimates obtained by repeated random subsampling (Figure 2; Figure 3). As the equations (5a) and (3) are mathematically congruent, *ρ *in the equations (5) and (5a) may be interpreted as a simplified empirical estimator for *θ*. The empirically derived regression coefficients *β*_*n*_, and *β*_*A *_would provide correction for possible deviations of the experimental population from the ideal population model.

We also compared allelic richness estimates obtained for the four conifer species using our regression model equation (5) and the rarefaction procedure. Rarefaction estimates were close to the subsampling and regression results obtained for *n *= 120 (Table 1). Rarefaction is commonly used to standardize allelic richness estimates to the smallest sample size used in a given study, but it cannot extrapolate the allelic richness beyond the values observed in the empirically analyzed samples [7]. Thus, it cannot be used for estimating the number of alleles in large populations. Our non-linear regression model is a good predictor for the allelic richness at large sample sizes. It effectively addresses the possible deviations from the ideal population model by introducing the empirically derived regression coefficients *β*.

**Figure 4:**
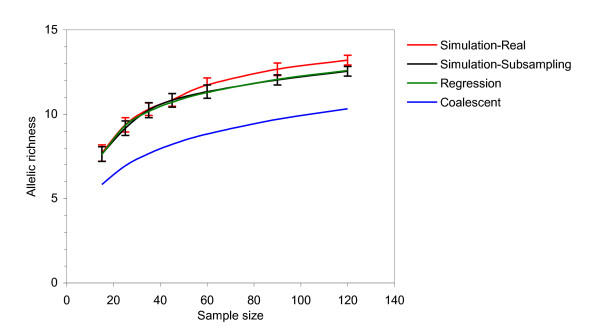
**Allelic richness estimates in the simulated data sets**. Simulation-Real - allelic richness observed in the total simulated data set A created by EASYPOP 2.1. Simulation-Subsampling - allelic richness observed in data set B, created by repeated random subsampling. Regression - allelic richness predicted by equation (5). Coalescent - allelic richness predicted by equation (3), *θ *estimated by coalescent from the data set B.

The proposed regression model developed in the present study has been validated by comparing the allelic richness parameters estimated by using different approaches in large Markov chain simulated populations (Figure 4). The allelic diversity estimated by our regression model was in agreement with that estimated for various sample sizes in the original simulated population of 10,000 individuals as well as that observed in subsampling of the 50 times amplified data for a subset of 200 individuals. However, the coalescent approach underestimated the allelic richness at all sample sizes (Figure 4).

A valid concern would be that the original sample set used for the subsampling procedure may contain only a fraction of the allelic diversity present in a large natural population. Our results indicate that allelic richness estimates obtained by the model developed here in the amplified data were consistent with that actually observed in the total simulated population. The allelic diversity of various samples drawn from the entire simulated population of 10,000 individuals (data set A) was consistent with that drawn from 50-times pseudo-replicated population of 200 individuals (data set B) (Figure 4). The replicated data sets based on *n *= 200 (data set B) adequately represented the major proportion of the allelic diversity existing in the entire simulated population (data set A), and pseudo-replication and subsampling apparently had little effect on allelic diversity estimates at the sample sizes below *n *< 100 typically used in population genetic studies. At large sample sizes (*n *> 500), the replicated data sets tend to underestimate the allelic richness in comparison with samples drawn from the true simulated population (Additional File 2). Capturing the low frequency (*p *= 10^-2^..10^-4^) alleles in a finite population would require sample sizes close to the entire number of individuals in the population.

It should be noted that existence of spatial genetic structure in a population can affect the observed allelic diversity estimate in a sample. In two of the four studied species, spatial genetic structure up to ~25 meters has been observed (Rajora, unpublished; Pandey and Rajora, submitted). Since the sampling distance normally used for population genetic studies in forest trees (30-50 m) is greater than the observed spatial genetic structure, the latter has little effect on the allelic richness estimates.

The logarithmic nature of the relationship between allelic richness and sample size holds true regardless of the organism and marker system used. In addition to our own data sets for conifer tree species, we observed this relationship in a number of other studies published for various taxa, e.g. [29-32]. In the present study, we provide a simple, direct and robust method to predict the allelic diversity in large natural populations. Leberg [6] mentioned one possible limitation of such extrapolation: it requires a significant number of samples for the initial estimation of *θ*, but in our opinion, the robustness of the subsequent results far outweighs the expenses associated with running a small pilot study.

Our approach takes into account possible deviations from the ideal population model occurring in such complex systems as natural forest tree populations, where long distance gene flow, population bottlenecks, selection, varying mating systems, and overlapping generations are the norm. One of the other advantages of our model over the coalescent approach is that it does not require high computation resources.

The minimum sample size for population genetics and conservation studies has been a hotly debated topic. Although it is usually desired to capture 90-95% of allelic diversity, it is often not feasible, as the true number of alleles in the population is rarely known. A recent study by Gapare and Aitken [29] claims that sample sizes of approximately 150 individuals per population would be enough to capture 95% of its alleles. However, the "true" number of alleles was observed at *n *= 200. Our simulation study and experimental results indicate that it is unlikely that a sample of 200 individuals would capture all alleles in a real natural population. The bivariate linear regression model used in [29] may not be an accurate predictor of allelic richness in large populations because the relationship between the sample size and the observed allelic richness is non-linear [6].

For conservation and adaptation studies, rare alleles may be especially important as they may represent the populations' potential to adapt in changing environmental conditions. Usually, very large sample sizes are suggested for conservation populations [1], although in our opinion the size of the conservation population can be optimized depending on the distribution of allele frequencies in the parent population. As the allelic richness is approximately a log function of the sample size, after certain threshold *n *(for example, *n *~150 in red spruce - Figure 1), the observed allelic richness increases almost exclusively by rare alleles. At a very large *n*, doubling the sample size would allow only a minor increase in the allelic diversity (Additional File 2). Sampling artifacts arising from the existing spatial genetic structure may further reduce the observed allelic richness increment. Our regression model could be a good predictor for the number of rare alleles in natural populations. Once the regression model parameters have been established for a given species and marker system, the results should be applicable for other populations within the species.

**Figure 5:**
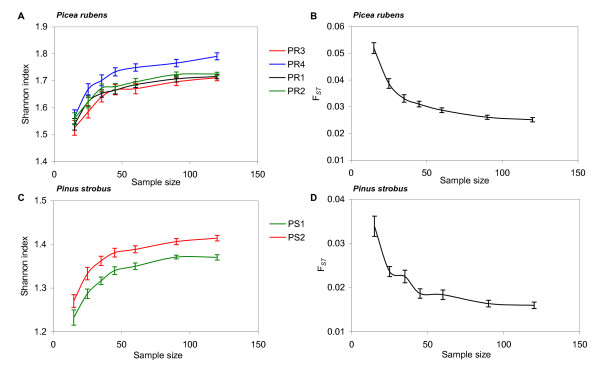
**Effects of sample size on Shannon diversity index and F_*ST*_**. A: Shannon index, *Picea rubens*; B: F_*ST*_, *Picea rubens*; C: Shannon index, *Pinus strobus*; D: F_*ST*_, *Pinus strobus*. Repeated random subsampling was performed on the empirical microsatellite data in 50 replicates. 95% confidence intervals are provided.

For most population genetic studies, an adequate sample size would be the one that allows for reliable estimation and comparison of genetic diversity and genetic subdivision parameters among populations. The effects of sample size on other observed population genetic parameters (observed and expected heterozygosities, F_*ST*_, Shannon diversity index) were illustrated using red spruce (*Picea rubens*) as an example. The observed and expected heterozygosities were generally insensitive to the sample size (data not shown), which corresponds well with the previously published data [8,9]. Shannon diversity index (Figure 5A; Figure 5C) and F_*ST *_(Figure 5B; Figure 5D) estimates were unstable at low sample sizes (*n *< 50), and stabilized after certain *n *(*n *= 60...90 in our case). Small allele frequency fluctuations have little effects on the observed F_*ST *_values. As discussed above, at large *n*, the observed allelic richness increases primarily by low frequency alleles that have little effect on the observed genetic differentiation parameters. Thus, *n *= 60 to 90 appears to be the optimum sample size for common population genetic purposes.

## Conclusion

Our non-linear regression model provides a simple and robust approach to estimate the genetic diversity in large natural populations based on the empirical data. Since the regression coefficients in our model are derived empirically, and there are no assumptions to violate, it allows for quick and easy estimation of allelic diversity in large natural populations based on finite sample sizes. The model is independent of the marker mutation mode and population history, and works well with high selfing and predominantly outcrossing species. It has been validated on simulated data sets, as well as on the experimental data for different species and molecular marker systems. Therefore, our model is more accurate, simple and practical than the coalescent or Ewens approach. The proposed method can be widely applicable in population genetic studies, and it may provide the missing link for conservation and management decision support.

## Competing interests

The authors declare that they have no competing interests.

## Authors' contributions

All authors contributed equally to the submitted work: SB generated the red spruce source data, developed the equation and drafted the manuscript; MP and OPR provided eastern white cedar, eastern white pine and white spruce empirical data, provided suggestions and revised the manuscript; and OPR is the Principal Investigator of the research program and provided funding and overall guidance and research directions. All authors have read and approved the final manuscript.

## Supplementary Material

Additional file 1 **An example of SAS NLIN input and output for estimating the regression coefficients of Equation (5)**. Click here for file

Additional file 2 Allelic richness estimated by repeated random resampling in simulated population genetic data with various combinations of migration and selfing rates. Click here for file

Additional file 3 **Allelic richness predictions for individual populations of all four species based on our regression model (5), Ewens formula and coalescent approach. **The population names are provided in Table 1. Regression - allelic richness predicted by equation (5). Ewens - allelic richness predicted by equation (3), *θ *calculated from the empirical source data set. Coalescent - allelic richness predicted by equation (3), *θ *estimated by coalescent from the empirical source data. Click here for file
