# Supplementary material for: A simple method for estimating genetic diversity in large populations from finite sample sizes
Source: BMC Genet. 2009 Dec 16;10:84. doi: 10.1186/1471-2156-10-84 (PMC2800116; doi:10.1186/1471-2156-10-84)
Supplement: Additional file 1 — An example of SAS NLIN input and output for estimating the regression coefficients of Equation (5). [file 1471-2156-10-84-S1.PDF]

Supplementary Material S1.

An example of SAS NLIN input and output for estimating the correction coefficients of Equation (5).

SAS program:

```
data WhitePine;
input
SampleSize GAP;
cards;
15     2.185333333
25     2.4
35     2.493333333
45     2.594666667
60     2.741333333
90     2.872
120    2.973333333

run;

Title WhitePine;

proc nlin data=WhitePine noitprint;

model GAP=rho*log(SampleSize+bN)+bA;

Run;
```

Model variables: rho = ρ; bN = β_n; bA = β_A

Output for White pine allozyme data, regression model (5)

The NLIN Procedure

NOTE: Convergence criterion met.

Estimation Summary

Method	Gauss-Newton
Iterations	3
R	9.214E-7
PPC(bN)	6.063E-6
RPC(bN)	0.014476
Object	9.13E-6
Objective	0.001426
Observations Read	7
Observations Used	7
Observations Missing	0

Source	DF	Sum of Squares	Mean Square	F Value	Approx Pr > F
Model	2	0.4548	0.2274	637.99	<.0001
Error	4	0.00143	0.000356		
Corrected Total	6	0.4562			

Parameter	Estimate	Approx Std Error	Approximate 95% Confidence Limits	
rho	0.3752	0.0340	0.2808	0.4695
bN	-0.5228	3.1469	-9.2598	8.2142
bA	1.1846	0.1612	0.7371	1.6322

Approximate Correlation Matrix

	rho	bN	bA
rho	1.0000000	0.9510422	-0.9969705
bN	0.9510422	1.0000000	-0.9679273
bA	-0.9969705	-0.9679273	1.0000000
