# Supplementary material for: A simple method for estimating genetic diversity in large populations from finite sample sizes
Source: BMC Genet. 2009 Dec 16;10:84. doi: 10.1186/1471-2156-10-84 (PMC2800116; doi:10.1186/1471-2156-10-84)
Supplement: Additional file 2 — Allelic richness estimated by repeated random resampling in simulated population genetic data with various combinations of migration and selfing rates. [file 1471-2156-10-84-S2.PDF]

Supplementary material S2. Allelic richness estimated by repeated random resampling in simulated population genetic data.

n	Data1: Nm=0; s=0		Data2: Nm=10; s=0		Data3: Nm=0; s=0.2		Data4: Nm=10; s=0.2		Data5: Nm=10; s=0.6		Data6: Nm=10; s=0.99		Data7: Nm=1; s=0.6		Data8: Nm=50; s=0.6		Data9: Nm=100; s=0.6		Data10: Nm=0, s=0.6	
	A	B	A	B	A	B	A	B	A	B	A	B	A	B	A	B	A	B	A	B
15	6.71±0.31	6.60±0.29	9.84±0.42	9.53±0.48	7.41±0.36	7.18±0.38	9.41±0.47	9.23v±0.41	7.70±0.48	7.63±0.44	6.56±0.46	6.78±0.43	8.85±0.50	8.49±0.63	10.01±0.51	9.30±0.62	10.34±0.61	9.97±0.54	7.79±0.46	7.63±0.44
25	7.82±0.35	7.49±0.34	12.70±0.43	11.12±0.52	8.62±0.37	8.32±0.35	11.11±0.44	10.85±0.46	9.37±0.42	9.16±0.43	8.06±0.48	8.26±0.50	10.57±0.51	10.21±0.54	12.08±0.48	11.39±0.57	12.51±0.49	11.69±0.53	9.26±0.39	8.91±0.43
35	8.44±0.39	8.14±0.42	12.69±0.39	12.17±0.38	9.35±0.37	8.77±0.36	12.12±0.40	11.87±0.45	10.30±0.37	10.24±0.45	9.02±0.44	8.42±0.45	11.60±0.45	11.22±0.51	13.21±0.48	12.69±0.36	13.77±0.51	13.09±0.54	10.12±0.45	9.78±0.34
45	8.93±0.38	8.50±0.38	13.41±0.34	12.67±0.40	9.90±0.34	9.30±0.35	12.87±0.40	12.56±0.40	10.85±0.38	10.80±0.41	9.48±0.51	8.86±0.45	12.22±0.45	11.81±0.44	14.07±0.41	13.39±0.46	14.53±0.51	13.78±0.42	10.58±0.34	10.22±0.34
60	9.67±0.36	9.14±0.42	14.11±0.39	13.50±0.34	10.51±0.33	9.82±0.33	13.63±0.41	13.16±0.38	11.73±0.42	11.34±0.39	9.82±0.45	9.22±0.44	12.73±0.38	12.46±0.38	14.93±0.39	14.13±0.40	15.49±0.37	14.67±0.40	11.22±0.40	10.78±0.29
90	10.57±0.37	9.81±0.35	15.11±0.34	14.34±0.35	11.29±0.30	10.43±0.25	14.57±0.32	14.03±0.31	12.66±0.37	12.03±0.30	10.48±0.41	9.54±0.35	13.61±30	12.97±0.39	16.05±0.37	15.09±0.40	16.47±0.38	15.54±0.35	12.10±0.32	11.40±0.29
120	10.99±0.31	10.10±0.28	15.61±0.34	14.80±0.28	11.79±0.34	10.75±0.30	15.13±0.33	14.50±0.30	13.20±0.29	12.54±0.28	10.88±0.46	9.64±0.37	13.96±0.27	13.48±0.28	16.68±0.36	15.61±0.32	17.11±0.25	16.09±0.28	12.62±0.34	11.80±0.25
500	13.43±0.34	11.53±0.12	17.65±0.28	15.82±0.12	13.64±0.31	11.11±0.13	17.13±0.28	15.88±0.15	15.13±0.26	13.69±0.13	12.02±0.32	9.98±0.12	15.00±0.14	14.15±0.09	18.60±0.21	17.02±0.14	18.69±0.18	17.37±0.15	14.75±0.29	12.98±0.15
2000	14.85±0.22	11.70±0.00	18.64±0.14	16.00±0.00	14.69±0.27	11.30±0.00	18.11±0.15	16.20±0.00	16.18±0.21	13.90±0.00	13.94±0.22	10.00±0.00	15.20±0.00	14.20±0.00	19.37±0.13	17.20±0.01	19.34±0.13	17.60±0.00	16.40±0.23	13.20±0.01
5000	15.61±0.19	11.70±0.12	18.82±0.10	16.00±0.00	15.54±0.19	11.30±0.00	18.44±0.16	16.20±0.00	16.84±0.20	13.90±0.00	15.04±0.18	10.00±0.00	15.20±0.00	14.20±0.00	19.65±0.09	17.20±0.00	19.58±0.09	17.60±0.00	17.14±0.22	13.20±0.00
10000*	16.20	11.70	19.00	16.00	16.30	11.30	18.80	16.20	17.60	13.90	18.40	10.00	18.80	14.20	19.80	17.20	19.70	17.60	18.00	13.20

Ten data sets were created using the EASYPOP 2.1 program (Balloux, 2001) under various migration (Nm 0, 1, 10, 50, 100) and selfing (s 0, 0.2, 0.6, 0.99) rates. Nm – migration rate, per generation, s – selfing rate. Allelic richness was estimated at sample size *n* by repeated random subsampling in the simulated (A) and replicated (B) data sets in 50 replicates. Standard deviations are indicated. *For *n*=10,000 the true number of alleles in the simulated population is provided.
