# Supplementary material for: A simple method for estimating genetic diversity in large populations from finite sample sizes
Source: BMC Genet. 2009 Dec 16;10:84. doi: 10.1186/1471-2156-10-84 (PMC2800116; doi:10.1186/1471-2156-10-84)

Supplementary Material S3.

Allelic richness predictions based on regression model (5), Ewens formula, and coalescent.

***Picea rubens* (PR) - microsatellites**

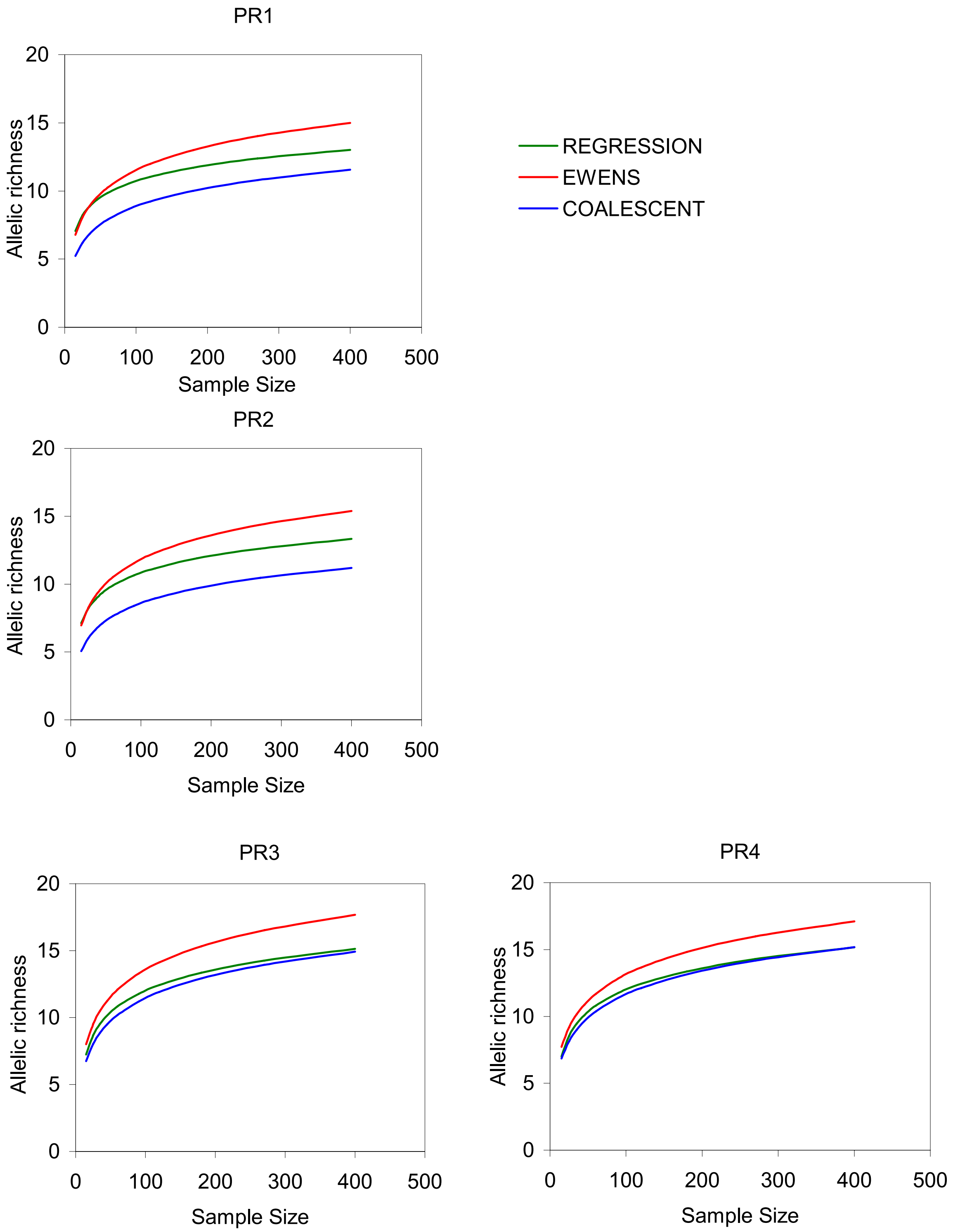

Thuja occidentalis (TO) - microsatellites

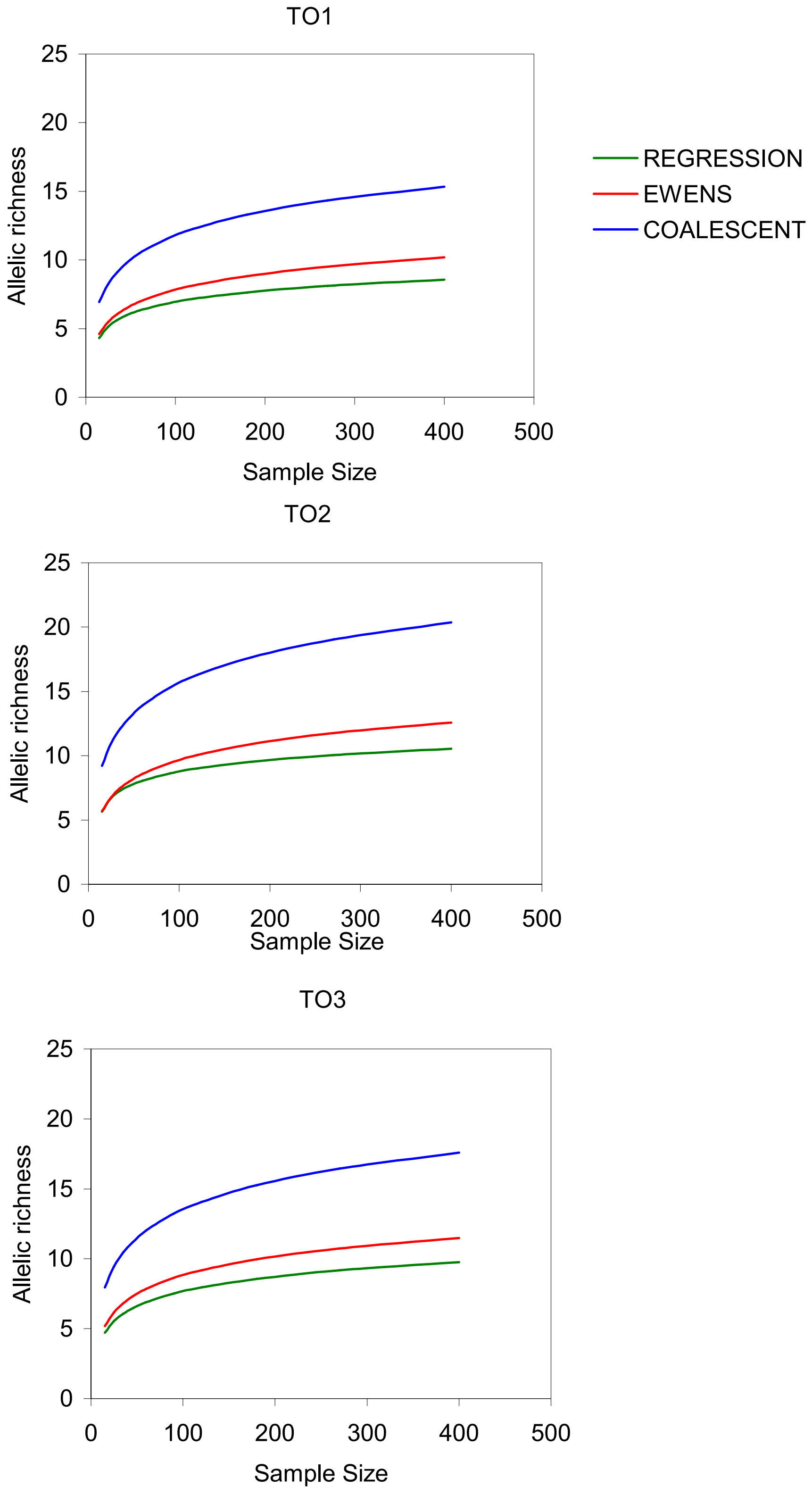

Picea glauca (PG) - microsatellites

PG1

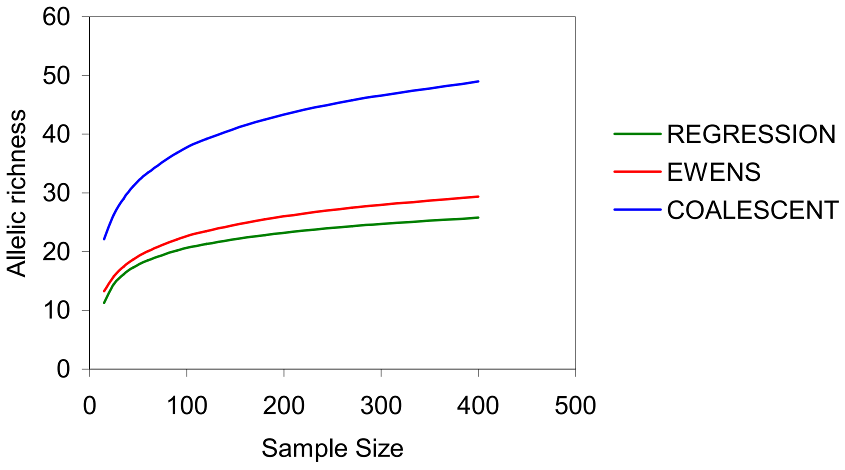

PG2

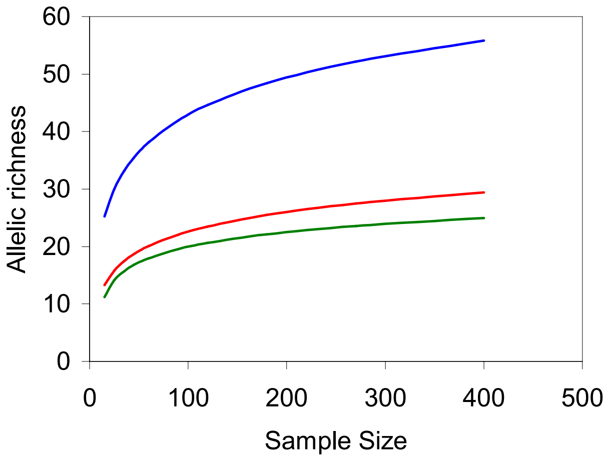

***Pinus strobus* (PS)**

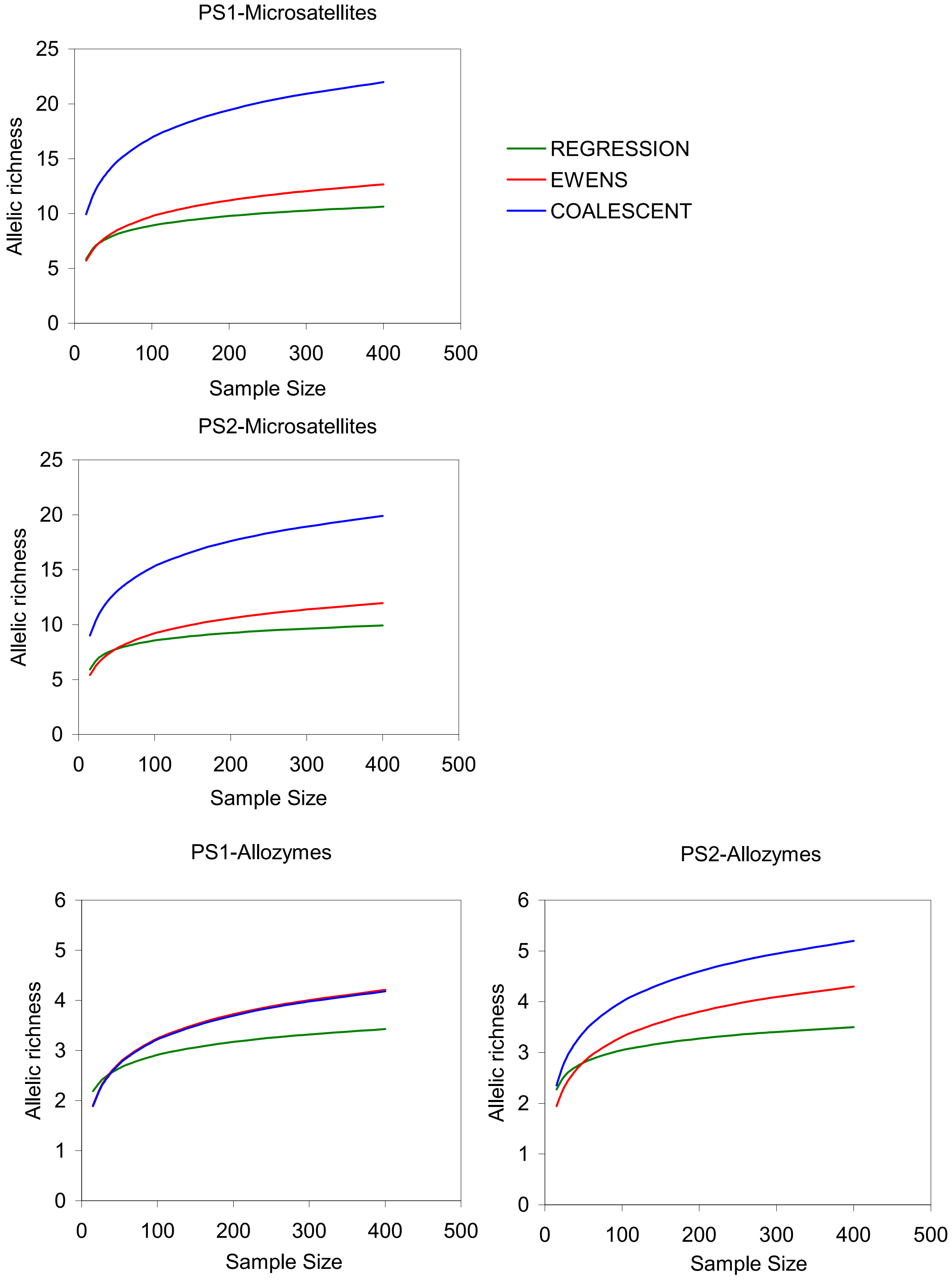

Supplement: Additional file 3 — Allelic richness predictions for individual populations of all four species based on our regression model (5), Ewens formula and coalescent approach. The population names are provided in Table 1. Regression - allelic richness predicted by equation (5). Ewens - allelic richness predicted by equation (3), θ calculated from the empirical source data set. Coalescent - allelic richness predicted by equation (3), θ estimated by coalescent from the empirical source data. [file 1471-2156-10-84-S3.PDF]
